# The oncogenic role of protein kinase D3 in cancer

**DOI:** 10.7150/jca.50899

**Published:** 2021-01-01

**Authors:** Yan Liu, Hang Song, Yehui Zhou, Xinxing Ma, Jing Xu, Zhenghong Yu, Liming Chen

**Affiliations:** 1 The Key Laboratory of Bio-Medical Diagnostics, Suzhou Institute of Biomedical Engineering and Technology, Chinese Academy of Sciences, Suzhou 215163, P. R. China.; 2 Jiangsu Key Laboratory for Molecular and Medical Biotechnology, Institute of cancer, Department of biochemistry, College of Life Science, Nanjing Normal University, Nanjing 210023, P. R. China.; 3 The First Affiliated Hospital of Soochow University, Soochow University, Suzhou 215006, P. R. China.; 4 School of Integrated Chinese and Western Medicine, Anhui University of Chinese Medicine, Hefei 230012, P. R.China.; 5 Department of Rheumatology and Immunology, Jinling Hospital, Medical School of Nanjing University, Nanjing 210002, P. R.China.

**Keywords:** Protein kinase D3, Cancer progression

## Abstract

Protein kinase D3 (PRKD3), a serine/threonine kinase, belongs to protein kinase D family, which contains three members: PRKD1, PRKD2, and PRKD3. PRKD3 is activated by many stimuli including phorbol esters, and G-protein-coupled receptor agonists. PRKD3 promotes cancer cell proliferation, growth, migration, and invasion in various tumor types including colorectal, gastric, hepatic, prostate, and breast cancer. Accumulating data supports that PRKD3 is a promising therapeutic target for treatment of cancer. This review discusses the functions and mechanisms of PRKD3 in promoting tumorigenesis and tumor progression of various tumor types as well as the latest developments of small-molecule inhibitors selection for PRKD/PRKD3.

## Introduction

**Figure 1:**
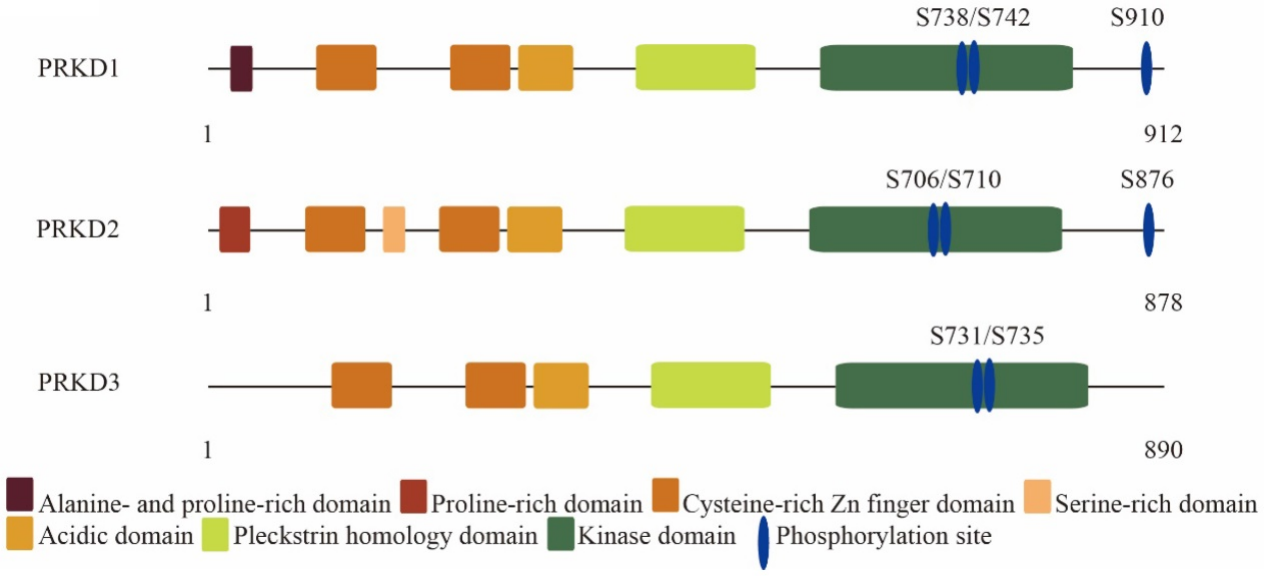
The molecular architecture of protein kinase D family members: PRKD1, PRKD2 and PRKD3.

Protein kinase D (PRKD) family consists of three highly conserved members in human: PRKD1, PRKD2, and PRKD3. PRKD1 was the first identified PRKD family member 1, 2. Two other PRKD members have since been identified, PRKD2 3 and PRKD3 4. PRKD family members are effectors of diacylglycerol signaling and are activated downstream of protein kinase C by a variety of stimuli including growth factors and hormones 5. PRKD family share similar structural features such as the highly conserved N-terminal regulatory domain containing two cysteine-rich DAG-binding C1 domains and an auto-inhibitory pleckstrin homology domain (**Figure 1**) 6, 7. Despite high structural homology among the PRKD isoforms, some structural variability exists and to a certain extent can help to explain the different effects of each PRKD isoforms. For example, PRKD1 and PRKD2 contain a c-terminal PDZ binding motif, while PRKD3 does not 8. The c-terminal PDZ binding motif allows PRKD1 and PRKD2 to regulate Kidins220 localization at the surface of neural cells and its trafficking between the plasma membrane and trans-golgi network, while PRKD3 do not have these functions 9. In addition, PRKD1 is mainly localized within the cytosol in resting cells 10, but upon stimulation can be found in other cellular structures such as the golgi 11, nucleus 12, or mitochondria 13. Like PRKD1, PRKD2 is mainly cytoplasmic in unstimulated cells 14. However, PRKD3 is localized in the cytoplasm and nucleus without stimulation 15. PRKDs have many cellular targets and have been implicated in a variety of biological effects such as cell growth 16, 17, invasion 18-20, angiogenesis 21, protein transport 22, transcriptional regulation 23, and epithelial to mesenchymal transition 24, 25. Due to lack of an autophosphorylation site at its C terminus and the alanine- and proline-rich region at PRKD3 N terminus, PRKD3 exhibits diverse biological effects and molecular signals from other PRKD isoforms in cancer. In this review, we focused on discussing PRKD3 in the context of cancer.

## Oncogenic functions of PRKD3 in breast cancer

Breast cancer is the most heterogeneous disease in females 26. PRKD1 is expressed and active in the normal breast ductal epithelial cells but its expression is lost during tumorigenesis. Analyses of human breast cancer specimens have shown that PRKD1 expression is completely lost in some of the most highly aggressive tumors 18, 27. However, PRKD2 and PRKD3 are only weakly expressed in the normal breast tissues while PRKD2 is generally weakly expressed, but PRKD3 has been reported to be up-regulated in breast cancer 28, 29.

PRKD3 is involved in all aspects of oncogenic signaling. Many researches have confirmed that PRKD3 is overexpressed in invasive breast cancer cell lines 28-30. Moreover, the mRNA and protein level of PRKD3 increased in triple negative breast cancer (TNBC) 28, 29. PRKD3 appears to have typical oncogenic effects in breast cancer. Depletion of PRKD3 attenuated cell proliferation by up to 40% in the TNBC cell line MDA-MB-231 31. In other TNBC cell lines (MDA-MB-468 and HCC1806) and in the ER-/HER2+ cell line (HCC1954), the same effect on cell proliferation was also observed 28. One of the possible mechanisms is that PRKD3 promotes TNBC cell proliferation via contributing to mammalian target of rapamycin complex 1/ribosomal protein S6 kinase B1 pathway activation 29. Other researchers suggest that PRKD3 promotes the proliferation of breast cancer cells by activating the mitogen-activated protein kinase 3/MYC proto-oncogene axis or ELAV like RNA binding protein 1 32, 33. The RhoGEF GRF-H1 is claimed to activate PRKD3 for the maintenance of TNBC stem cells 34. Besides functioning in proliferation of breast cancer cells, PRKD3 also promotes the motility, spreading, and migration of breast cancer. Basal PRKD3 activity promotes breast cancer migration via regulating cofilin phosphorylation status and activation of P21 (RAC1) activated kinase 4/LIM domain kinase 1 35. GIT arfGAP 1 phosphorylation on serine 46 by PRKD3 regulates paxillin trafficking and cellular protrusive activity 36. Knockdown of PRKD3 decreased the migration of ER- breast cancer cells with increased cell spreading and altered F-actin organization 28.

## PRKD3 participates in cell growth, invasion and secretion in prostate cancer

Besides breast cancer, increased levels of PRKD3 were detected in human prostate cancer specimens when compared to normal prostate specimens. In addition, there was a strong correlation between increasing prostate tumor grade and PRKD3 nuclear localization 37. PRKD3 promotes the growth and survival of prostate cancer cells through AKT serine/threonine kinase 1 and mitogen-activated protein kinase 1 signaling pathway 37. Interplay of PRKD3 with sterol regulatory element binding transcription factor 2 also contributes to the growth of prostate cancer cells via upregulating lipogenesis 38. Inducible silencing of PRKD3 inhibits secretion of tumor-promoting factors (matrix metallopeptidase 9, Interleukin 6, C-X-C motif chemokine ligand 8, and C-X-C motif chemokine ligand 1) in prostate cancer 39. Snail activated the lncRNA PCA3 expression could inhibit PRKD3 protein translation via competitive miR-1261 sponging to promote the invasion and migration of prostate cancer 40. PRKD3 promotes the invasion of prostate cancer cells by modulating nuclear factor kappa B subunit 1- and histone deacetylase 1-mediated expression and activation of plasminogen activator, urokinase 41.

## PRKD3 promotes cancer progression in other cancer type

PRKD3 acts as an important role as well as diagnostic criteria in gastric, melanoma, and hepatocellular cancer. In gastric cancer, PRKD3 promotes the development of cancer through RELA proto-oncogene, NF-KB subunit/6-phosphofructo-2-kinase/fructose-2,6-biphosphatase 3 activation of glycolysis 42. In melanoma cells, PRKD3 sensitizes RAF inhibitor RAF265 by preventing reactivation of mitogen-activated protein kinase 1 signaling 43. The expression of PRKD3 promotes the progression of hepatocellular carcinoma and predicts a poor prognosis in the patients with hepatocellular carcinoma after hepatectomy 44.

## PRKD3 inhibition in cancer therapy

The emergence of PRKD3 as a potential therapeutic target for various cancers has encouraged the development of potent, selective, and small-molecule inhibitors. Several small-molecule inhibitors such as 2,6-naphthyridine and bipyridyl and analogs 45-47, CID755763 and analogs 48, 3,5-diarylazoles 49, pteridine 50, CRT5 51, and CRT0066101 52 were reported to inhibit PRKD in various cell lines. An issue with most of these chemical compounds is that although they are effective in blocking cell growth, and migration* in vitro* 53, they are quickly metabolized when administered to xenograft mouse models. Among all these compounds only CRT0066101 has been used successfully in xenograft mouse models of colorectal 52, pancreatic 54, and breast cancer 55. In mice with TNBC, CRT0066101 significantly inhibited tumor growth without showing side effects 55. More importantly, the similar results were obtained with specific inhibition of PRKD3 suggesting that PRKD3 is CRT0066101's main target in TNBC cells 28. It is however possible, since CRT0066101 is administered orally, that some of the additional anti-cancer effects observed in the xenograft mouse model treated with CRT0066101 could be due to systemic inhibition of PRKD-mediated angiogenesis 56. CRT0066101 seems to be a promising candidate since no harmful effects have been observed in all the tested models. For breast cancer treatments that do not express PRKD1, pan- inhibitors of PRKD could be even more effective if used in combination with PRKD2 or PRKD3's current chemotherapeutic agents have been associated to mediate multi-drug resistance 43, 57. A potential problem with using pan-inhibitors is the management of detrimental off-target effects and to combat this problem the specificity of each compound must be fully investigated.

Alternative methods to chemical inhibition could include systemic delivery of siRNA or nucleic acid-based therapies that could allow to specifically target PRKDs 58, 59. Such strategies have been successfully used suggesting that the therapeutic applications could be very promising in humans 60. Last but not least, it would be promising to apply the new emerging proteolysis targeting chimera (PROTAC) for development of new drugs against PRKD3 for cancer treatment.

## Conclusion and perspectives

**Figure 2:**
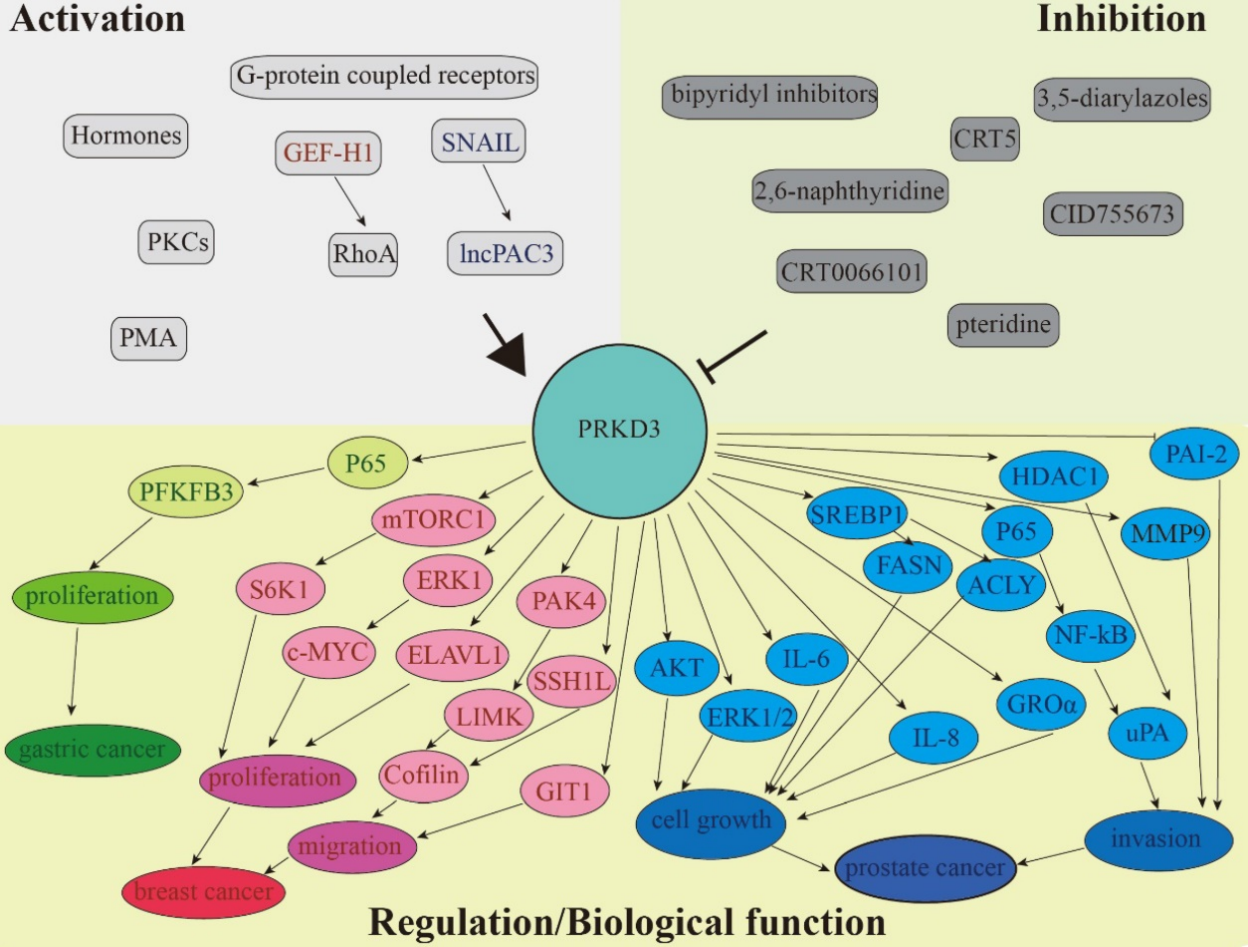
PRKD3 is involved in the regulation of various signaling pathways. Separate circuits show the ability of PRKD3 to promote cancer progression of gastric (green), breast (red), and prostate (blue).

There is a lot of evidence that PRKD3 is involved in the regulation of various signaling pathways, as well as in the integration of extracellular signals that promote migration, invasion, proliferation, and growth of cancer cells. This review summarized the various functions of PRKD3 in human tumors (**Figure 2 and Table 1**). Although many studies have confirmed the previously unknown mechanisms of PRKD3, it is still need for a better understanding of activation of different isoforms, isoform-specific functions, differential kinase expression and molecular cross-signaling. Delineation of potential compensatory effects between different PRKD subtypes in a specific cancer will help to improve the therapeutic prospects of PRKDs in a successful combinatorial molecular therapy approach.

## Figures and Tables

**Table 1:** The table shows the cancer-related functions of PRKD3 in a specific tumor type

Tumor type	Cancer-related function	Activation/regulation	Reference
Breast	Proliferation	TORC-S6K1/ERK1-c-MYC	29, 32
		ELAVL1	31, 33
	Migration	GIT1/PAK-LIMK-Cofilin	34, 35
		SSH1L-Coffilin	36
Prostate	Growth	AKT/ERK1/2/SREBP1-FASN	37, 38
		SREBP1-ACLY/IL-6/IL-8	39
		GROα	
	Invasion	P65/NF-ΚB-uPA/HDAC1-uPA	40, 41
		MMP9/PAI-2	
Gastric	Proliferation	P65-PFKFB3	42
